# Development and Applications of Superfolder and Split Fluorescent Protein Detection Systems in Biology

**DOI:** 10.3390/ijms20143479

**Published:** 2019-07-15

**Authors:** Jean-Denis Pedelacq, Stéphanie Cabantous

**Affiliations:** 1 Institut de Pharmacologie et de Biologie Structurale, IPBS, Université de Toulouse, CNRS, UPS, 31077 Toulouse, France; 2 Centre de Recherche en Cancérologie de Toulouse (CRCT), Inserm, Université Paul Sabatier-Toulouse III, CNRS, 31037 Toulouse, France

**Keywords:** fluorescent protein, superfolder, split-GFP, bipartite, tripartite, folding, PPI

## Abstract

Molecular engineering of the green fluorescent protein (GFP) into a robust and stable variant named Superfolder GFP (sfGFP) has revolutionized the field of biosensor development and the use of fluorescent markers in diverse area of biology. sfGFP-based self-associating bipartite split-FP systems have been widely exploited to monitor soluble expression in vitro, localization, and trafficking of proteins in cellulo. A more recent class of split-FP variants, named « tripartite » split-FP, that rely on the self-assembly of three GFP fragments, is particularly well suited for the detection of protein–protein interactions. In this review, we describe the different steps and evolutions that have led to the diversification of superfolder and split-FP reporter systems, and we report an update of their applications in various areas of biology, from structural biology to cell biology.

## 1. Superfolder Fluorescent Proteins: Progenitor of Split Fluorescent Protein (FP) Systems

Previously described mutations that improve the physical properties and expression of green fluorescent protein (GFP) color variants in the host organism have already been the subject of several reviews [1,2,3,4] and will not be described here. A centralized database with hundreds of fluorescent proteins (FPs) was recently developed (https://www.fpbase.org/), which provides researchers with easy access to up-to-date information [5]. In this chapter, we describe the most recent advances in superfolder protein engineering and their application in living cells and animals.

### 1.1. Improving the Folding of avGFP

The *Aequorea victoria* GFP or avGFP was cloned in 1992 by Prasher and colleagues [6]. It comprises 238 amino acids that adopt an 11-stranded barrel structure, known as “β-can”, with a central helix containing the chromophore [7]. The chromophore consists of three amino acid residues (Ser 65, Tyr 66, and Gly 67) that undergo cyclisation, dehydration, and oxidation during maturation. The maturation of the chromophore and therefore the emission of fluorescence requires proper GFP folding [7]. The auto-catalytic post-translational modifications of Ser 65, Tyr 66, and Gly 67 can occur when the protein is expressed in prokaryotic or eukaryotic species [8]. avGFP has a maximum excitation peak at 396–398 nm (corresponding to the neutral state of Tyr 66 in the chromophore) and a lower secondary peak at 476–478 nm (corresponding to the deprotonated anionic state of Tyr 66) [9,10], the emission peak is at 510 nm [9]. Thus, the excitation of avGFP by the 488 nm line of the Argon laser (standard in microscopy and flow cytometry) indicates low fluorescence intensity levels. avGFP was then evolved by site-directed mutagenesis to optimize brightness and excitation efficiency at 488 nm. The S65T mutation accelerates chromophore formation and promotes hydrogen bonds that stabilize the anionic state, resulting in a unique excitation peak at 489 nm and a brightness 5 times higher than that of avGFP [11]. In addition, the F64L mutation improves chromophore maturation at 37 °C [12]. Subsequently, the gene sequence of the corresponding S65T/F64L double mutant was converted to human codons to form the “enhanced-GFP” (eGFP), which combines a high expression rate in mammalian cells and a fluorescence intensity 30 times higher than that of avGFP [12].

**Figure 1:**
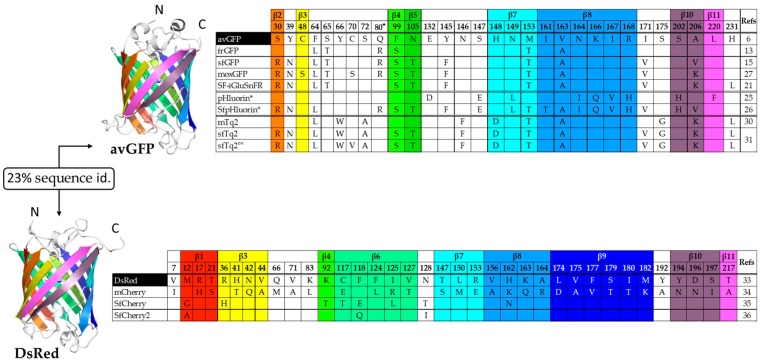
Superfolder variants of (top) green fluorescent protein (GFP) and (bottom) DsRed. The β-can structures of GFP (PDB code 1ema) and DsRed (PDB code 1g7k) share ~23% sequence identity. Strands β1 to β11 are colored from dark red to magenta in the ribbon structures and corresponding tables. Mutations refer to *A. victoria* GFP (avGFP) and DsRed indicated on a black background. The asterisk (*) refers to the ratiometric pHluorin. (^●^) The Q80R mutation is present in most cDNA constructs and may have resulted from a PCR error [19].

Recombinant avGFP produced in *Escherichia coli* is poorly soluble and even improved GFP variants like the folding reporter GFP or frGFP [13], which includes folding mutations F99S, M153T, and V163A [14] in addition to F64L and S65T [12], often exhibit folding defects when fused to other proteins. A molecular evolution strategy was applied to frGFP to create a superfolder GFP (sfGFP) variant whose folding is not affected by fusion to poorly folded proteins [15]. This new variant incorporates six mutations compared to frGFP (Figure 1) and is more resistant to chemical and thermal denaturation, with folding kinetics five times faster than that of frGFP [15] and ten times faster than that of the “Venus” GFP variant [16]. The high-resolution structures of frGFP and sfGFP highlighted a major role of the S30R mutation on the stabilization of the β-barrel through the formation of a network of electrostatic interactions [15]. Additional mutations have been identified that replace hydrophobic residues at the dimer interface with positively charged residues to force the monomerization of sfGFP [17] and other FPs for optimal performance as fusion tags [18].

### 1.2. Superfolder Color Variants and Applications

Experiments carried out on the extreme thermophile *Thermus thermophilus* (Tth), have shown that sfGFP, and not eGFP, could serve as a tool in cell localization experiments [20]. Given the exceptional stability of sfGFP, its tolerance to circular permutations was examined by cutting out loops connecting β strands while rewiring the original N- and C-terminal extremities. Circular permutants (cp) from sfGFP are mostly soluble with fluorescence yields higher than those measured from frGFP [15]. The most favorable cp provided a basis for testing protein insertion and creating genetically encoded sensors to study glutamate events in neurobiology [21], for cytosolic and cell surface ATP imaging [22], and neuronal activity [23]. The intensity-based glutamate-sensing fluorescent reporter (SF-iGluSnFR) was engineered from the *E. coli* glutamate-binding protein GltI and cp GFP [21]. In the case of the ATP sensor, cp-sfGFP is inserted between the two α-helices of the epsilon subunit of F_0_F_1_-ATP synthase from *Bacillus PS3*, a region predicted to undergo a large conformational change upon ATP binding [22]. This ATP sensor does not require exogenous substrate and may be complementary to existing luminescent luciferase-based reporters [24]. The cp-sfGFP voltage sensor, named accelerated sensor of action potentials 1 (ASAP1), is inserted in the extracellular loop that connects the third and fourth transmembrane segments of chicken (*Gallus gallus*) voltage sensitive phosphatase domain [23].

New GFP tools were required to answer specific questions relative to the influence of pH and oxidative environments. Two distinct groups of pH-sensitive fluorescent proteins sensors named pHluorins have been developed [25]. The ratiometric and ecliptic groups differ by the mutations they incorporate and their fluorescence excitation spectrum as a function of pH. Ratiometric pHluorin displays a reversible excitation ratio change at 395 and 475 nm between pH 7.5 and 5.5. On the other hand, the fluorescence intensity of ecliptic pHluorins excited at both wavelengths decreases with pH. At pH values less then 6.0, fluorescence is not detectable at 475 nm and barely detectable at 395 nm. Very recently, a superfolder variant of the ratiometric pHluorin was created for pH measurements in the endoplasmic reticulum (ER) of *Saccharomyces cerevisiae* [26].

Superfolder variants have also been created to address specific questions on the role and function of proteins in the periplasmic space of gram-negative bacteria. In such an oxidative environment, an ideal FP would lack cysteine in its sequence or be sufficiently robust to prevent artifacts mediated by the formation of disulfide bridges with negative impact on fluorescence intensities. Optimized FP variants for use in oxidizing environments, like moxGFP (Figure 1), were then created to overcome this limitation [27]. Surprisingly, mFruit color variants have no cysteine residue in their sequence [28] and they do exhibit differences in fluorescence brightness. On the other hand, sfGFP still folds and fluoresces in the periplasm of *E. coli* [29] despite the conservation of cysteine residues at positions 48 and 70. This would argue in favor of a more complex mechanism that also requires proper folding for periplasmic fluorescence. Based on mTurquoise2 [30], a superfolder variant named sfTq2 (Figure 1), which incorporates all six mutations of sfGFP, was created [31]. The advantage with bright cyan FPs donors is that they can accommodate a large palette of acceptors, either green, yellow, or orange, with high Förster resonance energy transfer (FRET) efficiencies. sfTq2 is now used in FRET experiments with acceptor mNeonGreen [32] for the detection of protein–protein interactions in cytoplasmic and periplasmic compartments. sfTq2 mutant C70V named sfTq2^ox^, when paired with mNeonGreen shows even brighter fluorescence signal in the periplasm [31].

With the discovery of the *Discosoma striata* mushroom anemone, which gave rise to tetrameric DsRed and optimized dimeric and monomeric variants [33], new variants have emerged with the aim of extending the color palette from orange to red and far-red. Starting with the monomeric fluorescent protein mCherry [34], a superfolder variant named SfCherry (Figure 1) was created by directed evolution [35]. The fact that sfCherry partition equally between monomers and dimers can lead to false positives in localization and interaction studies. A spacer-inserted sfCherry was used to improve the complementation efficiency between sfCherry1–10 and sfCherry11 with no further information on its oligomerization state [36]. This new variant named sfCherry2 incorporates three mutations: G12A, E118Q, and T128I (Figure 1). The engineering of bright, monomeric RFP variants has often been critical as mutations that disrupt the dimer interface can also affect fluorescence efficiencies. Recently a truly monomeric red fluorescent protein, named mScarlet, was created using the targeted multiple site-directed mutagenesis OmniChange method [37]. Although not described as a “superfolder” variant of RFP, mScarlet performs well as a fusion partner and in organelle labelling thanks to its high brightness, stability at low pH, and monomeric behavior [38].

## 2. Split-Fluorescent Proteins

### 2.1. Self-Associating Split-FP Systems

#### 2.1.1. Engineering of the Bipartite GFP1–10/GFP11 System

**Figure 2:**
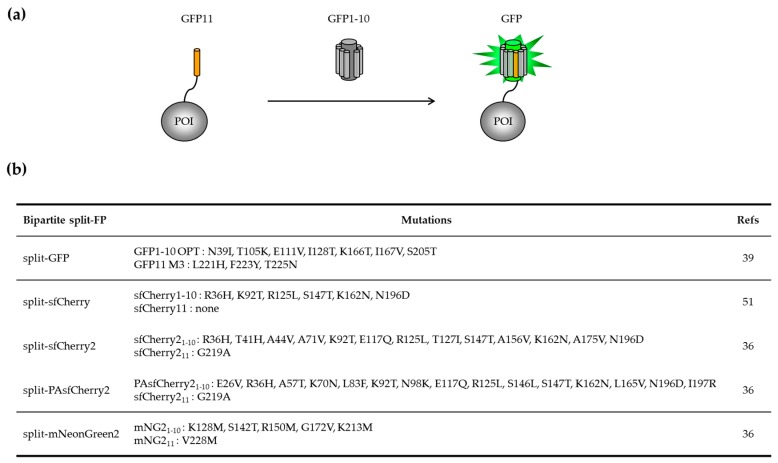
Self-associating bipartite split-FP systems. (**a**) Schematic representation of the first GFP-based detection system. The small GFP11 tag is C-terminally fused to the protein of interest (POI). Fluorescence recovery can only be achieved when GFP11 is accessible to complement the large fragment GFP1–10. (**b**) Known families of bipartite FP1–10/FP11 systems and mutations derived from sfGFP, sfCherry, and mNeonGreen [32].

The engineering of the first pair of GFP fragments that spontaneously associate without the intervention of protein partners was developed from sfGFP [39]. Using a random screen that consisted of cutting the loops that connect each β-strand of sfGFP and testing the capacity of each isolated β-strand to bind and restore fluorescence, one suitable cut-off site at position 214/215 was identified. Initially, the complementary fragment comprising strands β1 to β10, or GFP1–10, was not soluble when overexpressed in *E. coli.* Three rounds of directed evolution were performed on both GFP1–10 and GFP11 (strand β11) with the goal of increasing their solubility and kinetic of association. GFP1–10 OPT (optimal) and GFP11 M3 (mutant 3) are the basis of the bipartite split-GFP system that incorporates 10 mutations compared to sfGFP. The GFP1–10/GFP11 system requires the use of two compatible expression systems in vivo, a modified tetracycline-inducible pTET vector that carries the protein of interest (POI) fused to GFP11 fusion and an IPTG-inducible pET vector for the expression of the complementary GFP1–10 fragment [40]. Isolated GFP1–10 and GFP11 fragments do not fluoresce (Figure 2a). In situations where GFP11 is not accessible to complementation with GFP1–10, which may be due to poor folding or aggregation of the protein of interest, no fluorescence signal is detected. The analysis of 18 proteins fused to GFP11 M3 indicated no interference of the tag with the solubility of the passenger protein [39]. There is a perfect correlation between the amount of protein fused to GFP11 M3 and the intensity of fluorescence emitted after complementation, with subpicomolar detection range. The sensitivity of the “split-GFP” reporter to protein aggregation has led to the development of protocols that allow the quantification of soluble and insoluble fraction of expressed proteins labelled with GFP11 in a 96-well format [40,41].

#### 2.1.2. Applications of the Bipartite Split-GFP System to Monitor Protein Solubility In Vivo and In Vitro

Fluorescence measurements from the simultaneous expression of POI-GFP11 and GFP1–10 fragments corroborate with the total amount of protein expressed in cellulo, whereas fluorescence measurements in a sequential induction protocol only refer to the amount of soluble protein expressed [40]. Obviously this system is well suited for screening libraries of protein variants in *E. coli*, and to select optima for in vitro quantification of the soluble fraction [42]. This system was of key importance in confirming the increased solubility of the full-length mycobacterial enzyme PptT in *E. coli* compared to the N-terminal and C-terminal truncated versions. The addition of Co-enzyme A and Mg^2+^ ions to the cell lysate was essential to improve the stability of the enzyme in vitro [43]. The bipartite split-GFP can also be used in support to directed evolution protocols to normalize fluorescence values obtained from the screening of variants with improved enzymatic activities [44]. With the development of a fragmented approach called “domain trapping” (DT), it is also possible to identify soluble fragments of complex proteins that are difficult to study functionally and structurally in their entire form [45,46]. This high-throughput screening approach combines an ORF-filtering step with the GFP1–10/GFP11 system for the selection of clones expressing soluble fragments with single amino acid resolution boundary mapping.

The bipartite split-GFP technology can also be used to quantitatively measure aggregation of proteins in mammalian cells. For example, aggregation of the microtubule associated Tau protein as neurofibrillary tangles is found in Alzheimer’s and Parkinson’s diseases. The GFP1–10/GFP11 complementation system has enabled in situ monitoring and quantification of tau aggregation and the study of factors that modulate the aggregation process into living cells [47]. The system was also applied to the analysis of a series of naturally occurring and rationally designed variants of the pre-synaptic protein alpha-synuclein (αsyn), an intrinsically unstructured, misfolding-prone protein with a high propensity to form aggregates in Parkinson’s disease [48].

#### 2.1.3. Adapting the GFP1–10/GFP11 System for Protein Labelling in Mammalian Cells

The split-GFP system found its main applications in localization studies of target proteins in eukaryotic cells due to the small size of the GFP11 tag (15 amino acids) and the low background fluorescence. Recombinant GFP1–10 can be used as a reagent (not expressed directly in cells) to quantify POI-GFP11 in cell extracts or in fixed and permeabilized mammalian cells, either by microscopy or flow cytometry [49]. This application, called GFP1–10 complementation labelling, offers the advantage over traditional indirect immunodetection methods of having only one labelling step. Since the recombinant GFP1–10 solution is non-fluorescent and the fluorescence signal corresponds only to the specific complementation with the protein of interest, this method benefits from an excellent signal-to-noise ratio. Researchers have also used the inverse strategy to perform single-molecule imaging of proteins in living cells [50]. In the so-called method named complementation-activated light microscopy (CALM), the POI-GFP1–10 fusion becomes fluorescent upon external addition of the synthetic GFP11 peptide to the cells. In addition, labeling with GFP11 allows highly specific tracking of individual molecules for long periods of time due to the irreversibility of the complementation. It also offers the possibility to perform dual tracking of complemented GFP with an acceptor peptide in FRET experiments [50]. More recently, Bo Huang and colleagues made a series of significant progress in the exemplification of split-GFP applications in the field of endogenous protein labelling. In a first study, the authors have shown that introducing GFP11 into genomic loci via the CRISPR Cas9 system enables the visualization of endogenous proteins [51]. The detection of proteins with low levels of endogenous expression was improved by introducing several repeats of the GFP11 tag. It is interesting to note that the photobleaching rate of the reconstituted GFP is slower than that of the full-length GFP and decreases significantly with an increase in the number of GFP11 copies. The mechanism of this reduced photobleaching is still unexplained, and it cannot be attributed to the reversibility of the split-GFP association even in live cells. In another study, the same investigators have demonstrated that this strategy can be scalable to higher throughput by targeting 48 human genes in parallel in proteomics studies [52].

With the development of new color variants, additional split-FP pairs have been engineered (Figure 2b). This is the case of the split-Cherry variant [51] developed on the basis of sfCherry [35]. Huang and colleagues also described a simplified engineering strategy that consisted of inserting a long flexible spacer at the cut site between strands β10 and β11 and evolving such fusion for improved brightness. Such an approach successfully led to the development of sfCherry2 (Figure 1) and mNeonGreen2 that served as a basis to create split-FP fragments [36]. These new split-FP systems have been successfully used in dual color labeling of endogenous proteins at specific locations of the endomembrane system [36].

#### 2.1.4. Applications in Cell Biology

It is conceivable to pre-locate the GFP1–10 detection fragment in order to exploit the spatial nature of the detection. Thus, only a fluorescence signal will identify the transfer of proteins to another cell or to a different compartment within the same cell. Numerous studies have exploited these features of the split-GFP approach to detect proteins in specialized subcellular compartments of mammalian cells. The most widespread applications involve neuronal cell communication, subcellular protein localization, and host–pathogen interactions.

##### Neuronal Cell Communication

Cornelia Bargmann’s laboratory (Rockefeller, NY) first exemplified the use of the GFP1–10/GFP11 system to detect synaptic connection partners in ***Caenorhabditis*** *elegans* [53]. The method named GRASP (mammalian GFP reconstitution across synaptic partners) monitors the fluorescence of the reconstituted GFP following the recognition of synaptic partners fused to split-GFP domains. Since then, this approach has become popular in the field and was extended to Drosophila [54,55] and mice [56]. This area of research also benefited from the development of yellow and cyan variants of the bipartite split-GFP system by simply mutating GFP1–10 OPT for in vivo co-labelling experiments [57].

##### Protein Topology and Subcellular Localization

The dissociation of GFP into two domains enables different tagging approaches and may specifically address the topology of proteins in organelles. GFP1–10 can be diffusely expressed in the cytoplasm or fused to an inner component of the ER such as calnexin to determine if the POI-GFP11 fusion is located in the lumen or at the surface of the organelle [58]. Cytosolic expression of GFP1–10 was also used to visualize the process of dislocation of ER proteins where poorly folded proteins are transported to the cytosol before degradation by the proteasome [59,60]. A similar approach was adapted to monitor the translocation of the DJ-1 protein and Parkinson disease-associated mutants at the mitochondrial outer membrane and the matrix in response to cellular stresses [61]. Recent use of this tagging method allowed the analysis of contact sites between organelles and the identification of organelle tethering structures between the ER and mitochondria [62,63,64]. In the same way, various GFP11-mCherry reporters were designed to specifically detect proteins at the inner nuclear membrane (INM) in yeast. A library of transmembrane proteins in fusion with GFP1–10 was generated to identify proteins localizing at INM. Thorough validation of candidates confirmed the specificity of split-GFP assay that defined a set of 400 components of the INM proteome [65]. Adaptation of the split-GFP assay to address topology in membranes was also described in plants [66].

##### Host–Pathogen Interactions

The dissociation into two non-fluorescent fragments that reassemble spontaneously is a method of choice for the study of translocation events between a pathogen and the host cell. The GFP1–10/GFP11 system has made it possible to visualize the location and to characterize the dynamics of viral proteins [67], bacterial proteins [68], or parasitic proteins addressed to different subcellular compartments of the host cell [69]. These applications were recently the subject of a detailed review [70]. Amy Palmer’s laboratory first demonstrated the possibility to visualize the internalization of Salmonella effector proteins fused to GFP11 into a host cell that expresses the GFP1–10 fragment [68,71]. The authors lately extended the GFP1–10/GFP11 approach to visualize the infection of epithelial cells with Listeria virulence proteins using split-FP systems derived from mNeonGreen and sfCherry, providing a set of various tagging alternatives to perform multicolor imaging of bacterial infection [72]. The group of Pan developed the assay to follow the internalization and the intracellular trafficking of virulence factor VirE2 from *Agrobacterium tumefaciens* in plants [73,74].

### 2.2. Protein–Protein Interactions

The detection of protein–protein interactions (PPIs) in their cellular context is key to identify molecular mechanisms in cell signaling pathways and elucidate dysfunctions of cellular processes. One of the most widespread genetic approaches for the analysis of PPIs in cells is the yeast two-hybrid (Y2H) system, which involves the reconstitution of a transcription factor that drives the expression of a reporter protein [75,76]. Y2H detection systems and derivatives have brought an important contribution to the elucidation of protein interaction networks due to their capacity of performing genome-wide screening assays. The main limitation of 2H assays resides in the inability to localize the complex formed. Bimolecular fluorescence complementation (BiFC) relies on the complementation of two non-fluorescent hemi-GFPs to form a functional GFP following the interaction between two protein partners fused to them [77,78]. BiFC allows the direct visualization of protein complexes and their intracellular trafficking in live cells. With the advent of multicolor FP variants, monitoring the association between interacting partners has never been more accessible [79,80]. A detailed list of available BiFC pairs was recently published and will not be further discussed here [4,81].

A major advantage of split-FP systems is that they do not require additional substrates or cofactors, and are particularly easy to implement, by simply measuring a fluorescence signal. Because assembly of the FP fragments is irreversible, the stability of the BiFC enables detection even in the case of transient interactions and low affinity complexes [82,83]. One major disadvantage with BiFC is the poor solubility of the split-FP fragments, which may interfere with protein association [84]. Adaptation of the BiFC split-fragments to the frGFP and sfGFP variants, whose solubility is improved compared to the parental split-Venus system [79], resulted in high fluorescence levels in the absence of fused protein partners due to self-assembly of the hemi-domains [85]. Therefore, improving BiFC solubility induces self-association of FP domains and its inability to function as a protein-protein interaction reporter.

#### 2.2.1. Engineering of a Tripartite Split-GFP System

**Figure 3:**
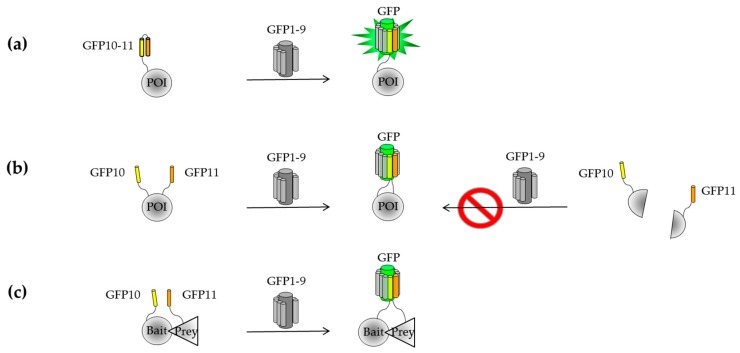
Stepwise engineering of a tripartite split-GFP protein–protein interactions (PPI) detector. (**a**) Bipartite self-system based on GFP10–11/GFP1–9 association. (**b**) Bipartite self-association system based on a sandwich model GFP10-POI-GFP11 where strands β10 and β11 are positioned at the N- and C-terminus of the POI, respectively. The proximity of strands β10 and β11 allows the association with GFP1–9. A truncated POI leads to GFP10 and GFP11 fusions that are unable to associate with GFP1–9 (right image). (**c**) Interactions between bait and prey proteins bring GFP10 and GFP11 closer in space for complementation with GFP1–9.

To overcome inherent self-assembly issues occurring with BiFC and the GFP1–10/GFP11 systems, a new assay that relies on the association of three split-FP domains (tripartite) was developed [85,86]. The implementation of such approach required a stepwise engineering of a two-body self-associating split-GFP pair consisting of a large GFP fragment (strands β1 to β9, or GFP1–9) and the C-terminal domain of sfGFP that includes the β-hairpin structure formed by strands β10 and β11. These individual fragments are not fluorescent on their own, but spontaneously associate when co-expressed to reconstitute the full-length GFP (Figure 3a). Molecular evolution of both GFP1–9 and the GFP10–11 hairpin domain was first used to improve their solubility as stands alone fragments and in the context of a GFP10-POI-GFP11 fusion (Figure 3b). Interestingly, fluorescence measurements of *E. coli* cells expressing GFP1–9 and GFP10-POI-GFP11 correlate with the amount of soluble protein expression, similarly to the GFP1–10/GFP11 system [85]. Such a topology may be beneficial in the case of truncation artifacts and translation reinitiation sites (IRBS) arising from mutagenesis methods with single base substitution (Figure 3b). This was the starting point for creating a tripartite split-GFP system composed of GFP10 (residues 194–212) and GFP11 (residues 213–233) fused to the bait and prey proteins, respectively (Figure 3c). In the absence of interactions, the large complementary fragment GFP1–9 (residues 1–193) cannot complement the distant GFP10 and GFP11 tags, while interacting partners will bring together GFP10 and GFP11 for GFP1–9 complementation to reconstitute a functional GFP. Each fragment of the tripartite system has been engineered independently to eliminate interferences with fusion proteins. The GFP11 domain, named GFP11 M4, has two substitutions and different split boundaries compared to its predecessor GFP11 M3 [39]. The variant GFP1–9 OPT includes four additional substitutions compared to GFP1–9 M1 that enable the detection of proximal strands β10 and β11 [85].

To further confirm the reliability of the tripartite split-GFP system, GFP10 and GFP11 were fused to lysine-rich (K) or glutamate-rich (E) coiled-coil domains to favor interactions between opposite charge coiled-coil domains or repulsion in the case of same charge coiled-coil domains [87]. Assays using purified recombinant GFP10-K/E-GFP11 indicated a detection limit in the subpicomolar range [85]. Moreover, no background fluorescence signal was detected for repellent GFP10-E/E-GFP11 pair, thus demonstrating that there is no non-specific association between GFP tags in the absence of interaction between E domains. These model systems validated the robustness and the specificity of the method for in vitro and in vivo studies. The small size of the GFP10 and GFP11 tags does not perturb the soluble expression of passenger proteins in *E. coli* and enables the analysis of protein association in vitro. The position of GFP10 and GFP11 at the N or C-termini of candidate proteins still depends on protein conformation and topology. Increasing linker length from 12-mer to 25-mer allows adapting the system to larger proteins and multi-protein complexes [85]. Although highly specific, low amounts of the detection fragment GFP1–9 may result in lower yield and complementation efficiency. The conditional association of FKBP12 (human FK506-binding protein FKBP) and FRB (FKBP12-rapamycin-associated protein) with rapamycin (RAP) [88] was used to characterize GFP1–9 binding and association. Detection of a fluorescence signal following the formation of the RAP/FRB/FKBP12 complex was detected 15 min and 30 min after addition of RAP in vitro and in mammalian cells, respectively [39]. This is in accordance with the time intervals recorded for previously described BiFC systems [89]. As commonly observed with protein–protein interaction reporters, dose-response binding curves of FRB/FKBP association with rapamycin indicated that the observed association rates from split-GFP complementation are in good agreement with dissociation constants determined using other techniques such as surface plasmon resonance (SPR).

#### 2.2.2. Known Examples of Tripartite Split-GFP Applications in Cell Biology

##### Screening of Novel Inhibitors

Like all protein complementation assay (PCA) developed so far [90], the tripartite split-GFP system is suitable for small molecule screening. Although irreversible, this system could detect the abolition of rapamycin-induced FRB/FKBP association in the presence of the FK506 competitive inhibitor [85]. Recent reports describe a few examples of applications of the tripartite system for PPI inhibition screening. Zhang and colleagues recently developed a biosensor that makes use of GFP10 and GFP11 peptides fused to the sortase A (SrtA) recognition sequence and oligoglycine, respectively, so that sortase activity mediates transpeptidation prior to tripartite complementation [91]. Two potent inhibitors of SrtA were used as positive controls to validate the use of the tripartite system for high-throughput drug screening [91].

**Figure 4:**
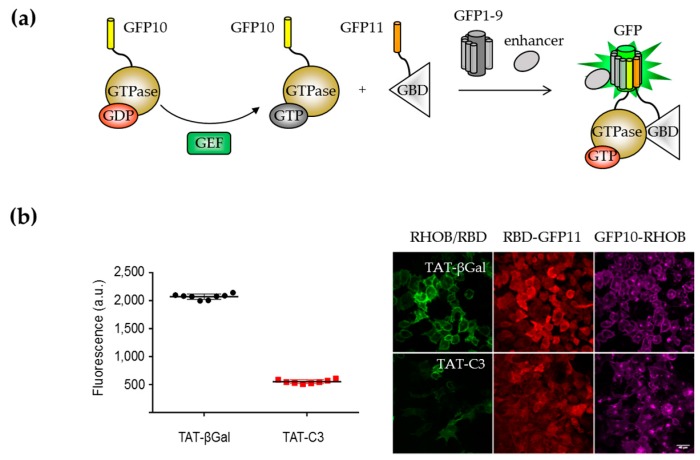
Analysis of the modulation of small GTPase activation with the tripartite split-GFP system. (**a**) When the GTPase is activated by guanine exchange factors (GEFs), the GTPase associates with the GTPase binding domain (GDP) of an effector protein. A VHH antibody that recognizes the full-length GFP is integrated in the cell line to enhance the fluorescence of reconstituted GFP. (**b**) Fluorescence analysis of RHOB activation reporter cells treated with 5 μg/mL of TAT-C3 RHO inhibitor or with TAT-βGalactosidase control. *n* = 8 replicates of one experiment. Right image representative wide-field images showing the GFP fluorescence of RHO/RBD (rhotekin binding domain), and immunostaining with GFP10 antibody (GFP10-RHO) and GFP11 antibody (RBD-GFP11). Scale bar: 40 μm.

High-content screening (HCS) assays in mammalian cells using the tripartite split-GFP system have been developed for the detection of modulators of small GTPase activation [92]. In these experiments, the split-GFP system is used to monitor the association between active GTP-bound GTPases and their effectors, under the control of guanine nucleotide exchange factors (GEFs) (Figure 4a). A quantitative high-throughput 96-well plate fluorescence assay was developed from stable cell lines models that reports RHO activation following the interaction of RHOB GTPase with the RHO-Binding domain of its effector rhotekin (RBD). Proof of principle of the assay was demonstrated using a cell permeable version of C3 exoenzyme (TAT-C3) that inhibits RHO activation by ADP-ribosylation [93]. Cells incubated with TAT-C3 indicated a 75% decrease of reporter fluorescence intensity compared to control cells (TAT-βGal). Specific antibodies to GFP10 or GFP11 tags have been developed to detect GFP10- and GFP11-tagged chimera in combination with the analysis of the RHO/RBD split-GFP complex (Figure 4b). Overall, these results demonstrate that the tripartite split-GFP system is a robust tool to detect early events of GTPase activation. The accumulation of the split-GFP signal provides a high dynamic range and a flexible time detection window for the high-throughput screening of inhibitors. The development of such a system opens of the route to the study of small GTPase activation mechanisms including regulators and upstream mediators of small GTPases signaling. Obviously, this approach may have interesting applications beyond the field of small GTPases to evaluate the effect of chemical and pharmacological compounds on their association of various signaling modules.

##### Monitoring Direct Associations

As seen with GFP1–10/GFP11, the tripartite split-GFP system is sensitive to protein folding and solubility issues that can interfere with complementation. This system was used to identify a new functional oligomerization scenario for TDP-43, a DNA-binding protein that mislocalizes in the cytoplasm where it forms insoluble aggregates associated with neurodegeneration. Fusion of GFP10 and GFP11 to the N- or C-terminal regions of adjacent TDP-43 was key to elucidate their relative positioning within oligomers [94]. Although the tripartite GFP11 tag differs from the bipartite system by two amino-acid substitutions and different lengths, both tags may be used in the context of BiFC and TriFC (trimolecular fluorescence complementation) experiments [85]. These two split-GFP technologies have been used to study the localization and self-assembly of TDP-43 [95]. In another study, Wei and colleagues studied the dimerization of the receptor for advanced glycation end products (RAGE) [96]. Unstable RAGE dimers form via non-covalent cysteine bonds in the ER and are subjected to ER-associated degradation. Direct interactions within the 10-subunit transcription factor IIH (TFIIH) were investigated in the context of the wild-type and patient-mutated Trichothiodystrophy group A (TTDA) protein variants [97]. Fluorescent recovery after photobleaching (FRAP) experiments on the reconstituted GFP complex showed binding of the complex to DNA and its recruitment to the DNA damaged region. The tripartite split-GFP system has also been adapted to yeast in an attempt to elucidate the organization of septins that form rod-shaped complexes and higher-order structures in the cell cortex [98]. The authors exploited the modularity of the system by varying the positions of GFP10 and GFP11 and exploring different linker lengths to identify the topology of septin complexes [98]. Recently, the tripartite system was applied to the detection of membrane proteins association in planta [99]. The system was modified to incorporate a dual-intein domain that allows synchronizing protein expression of the tripartite split-FP components.

##### Monitoring Indirect Associations

**Figure 5:**
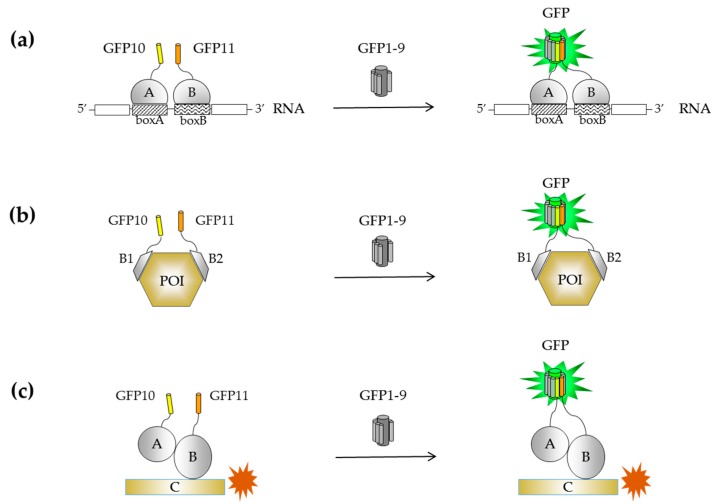
Tripartite split-GFP as a proximity assay tool to monitor indirect associations. (**a**) Two proteins (A and B) that bind RNA at specific target sites (boxA, boxB) are fused to GFP10 and GFP11. RNA sequences were designed to contain box A and box B in tandem (box AB), connected by a five-nucleotide linker. Binding of proteins to their target sequence forms a complex that is detected upon addition of GFP1–9. (**b**) Two affinity binders (B1 and B2) of a protein of interest (POI) are fused with GFP10 and GFP11. GFP reconstitution allows quantification of binding efficiency of B1 and B2 to their target. (**c**) The trimeric complex A-B-C is detected by co-localization of the complex formed by A/B and GFP, and the third binding partner C fused to a red fluorescent protein marker.

The modularity of the single-strand tagging system has found applications in protein engineering that relies on the detection of multiple protein complexes, as exemplified with the heterotrimer *Tus BCD* complex (YheNML) [85]. One key advantage of this proximity sensor is in the length of the linkers between the protein of interest and the GFP tags that must be adapted to optimize complementation. Proximity of two RNA-binding proteins tagged to GFP10 and GFP11 was used in a RNA detection assay [100], which shows an excellent signal-to-background ratio (70-fold) in vitro (Figure 5a). The same authors implemented the tetramolecular fluorescence complementation (TetFC) method in *E. coli* to isolate cells expressing the target RNA by FACS [101]. The tripartite system has also been applied to measure the binding affinities of small non-immunoglobulin scaffolds that bind to a target protein at two non-overlapping epitopes [102]. Here, only the binders are tagged with GFP10 and GFP11, and recognition of the target protein is monitored from fluorescence readings (Figure 5b). In a study conducted by Polge and colleagues, a combination of yeast two- and three-hybrid screens, SPR, and split-GFP approaches were used to characterize trimeric complexes formed by E2/MuRF1 ubiquitin enzymes and their substrate telethonin [103]. Tripartite split-GFP assays detected the association of identified E2/MuRF1 complexes with affinities in the submicromolar to nanomolar range. The trimolecular association of MuRF1-E2-telethonin was further confirmed in cells with the relocalization of mCherry-telethonin to the perinuclear region where GFP-MurF1-E2 complex localized (Figure 5c).

##### Biosensor Design

A protease sensor named FlipGFP was recently designed by inverting the orientation of strand β11 with respect to strand β10 so that complementation with GFP1–9 can no longer occur [104]. Insertion of a protease cleavage site reverts the orientation of β11 after protease digestion to form an antiparallel structure that binds GFP1–9 and restore the fluorescence. The biosensor displays a 100-fold fluorescent change upon TEV protease cleavage and is sensitive to the activation of endogenous caspase-3 mediated apoptosis, in correlation with the induction of caspase-3 activity. The caspase activity sensor was applied to live cell animals, zebrafish, and Drosophila. A less sensitive red version of the protease reporter was engineered from sfCherry by the same authors [104].

## 3. What’s Next in the Split-FP Development?

### 3.1. Extending the Spectra of Split Fluorescent Protein Detection Systems

Extending the emission spectrum of FPs in the range of 650 to 720 nm would have a considerable impact in PPI application in vivo thanks to a low absorption in tissues and reduced background noise at these wavelengths. As a consequence, only a limited number of split-GFP applications have been reported in mice. One study reported a BiFC system engineered from the far-red monomeric fluorescent protein mNeptune with an excitation peak at 600 nm and an emission peak at 650 nm that was used to visualize PPI in live cells and mice [105]. The system was shown to detect weak dimerization association between EGFP dimers with good signal-to-noise ratios, using control leucine zipper heterodimerization in live cells. Interactions between influenza viral NS1 protein and the 5′-untranslated region (UTR) of nucleocapsid protein (NP) messenger ribonucleic acid (mRNA) and matrix protein (M) mRNA were detected from BiFC and TriFC experiments on mRNA–protein interactions. TriFC signals could be observed in live mice without eliminating autofluorescence signals from tissues.

Novel classes of GFP-unrelated FPs with far-red fluorescence signal were recently developed. These systems are not fluorescent on their own, but switch on when covalently coupled to a nucleotide such as biliverdin [106] or phycocyanobilin [107]. Recently, a BiFC system was developed from iRFP720 by molecular evolution of iRFP, a bacterial phytochrome that is expressed in its monomeric form and that does not require a co-factor apart from endogenous biliverdin [108]. A proof-of-principle of the use of this variant as a PCA system has been reported for the detection of PPI in live cells and mice [108]. Note that all these far-red BiFC have been tested with model proteins, and not extensively characterized.

Two reversible BiFC systems have been reported from FPs that require exogenous chromophores. IFP1.4 is a monomeric biliverdin-containing bacteriophytochrome FP that was engineered into a reversible PCA for the study of the dynamics of protein complexes in living cells [109]. Its main disadvantages are its low quantum yield and poor brightness, which limit its applications for protein complex localization studies. unaG is an FP derived from Japanese eel that incorporates endogenous bilirubin as the chromophore. A BiFC assay derived from unaG, named UnaG-based PPI reporter (uPPI), exhibits a faster kinetic complementation rate [110]. Further characterization and improvement of these systems may trigger new applications for visualizing reversible processes.

### 3.2. Improving the Maturation Rate of the Chromophore After Complementation

#### 3.2.1. Engineering of Detection Fragments for Improved Conformational Homogeneity

Kinetic studies of interactions between GFP11 M3 and GFP1–10 have revealed that GFP complementation is not limited by the rate of association of split-GFP fragments but rather depends on the de novo maturation of the chromophore [39]. Still, a better understanding of the folding of GFP1–10 in vitro could help improving this self-assembly process. GFP1–10 was shown to partition between monomeric and dimeric forms [39,111]. Further in-depth analysis of the assembly of these split-GFP fragments indicated that the oligomerization state of GFP1–10 is important for association with GFP11, suggesting a more favorable binding of GFP11 to monomeric GFP1–10 [111]. Therefore, we lack information on how the design of a monomeric GFP1–10 variant could improve the kinetic properties of the split-GFP association. Similar investigations should apply to GFP1–9.

#### 3.2.2. Prematuration of the Chromophore for In Vitro Assays

Split-GFP association is a slow but irreversible process. Dissociation of split-GFP fragments can only be achieved in vitro by photodissociation of the reconstituted barrel [112] or chemical disruption of peptide bonds in denaturation conditions [113]. Renaturation of isolated split-GFP fragments induces the reassociation of the two fragments into a functional GFP molecule, with a five-time increase in fluorescence intensity [114,115]. Following these observations, several studies have exploited the prematuration of the chromophore in order to improve split-GFP kinetics. These include the production of prematured version of GFP1–10 or GFP1–9 by incorporating a proteolytic site in the GFP scaffold that release strand β11 [113,114] or β10–11 [116]. Alternative approaches involve coexpressing both fragments and inducing their disassembly under denaturation conditions [114], or purifying each fragment separately under acid-pH conditions [115].

#### 3.2.3. Use of Scaffolds Binders to Increase the Stability of the Chromophore

Another alternative to improve split-GFP fluorescence in live cells is to use GFP binders that modulate the maturation and brightness of the GFP chromophore. One single-domain antibody based on camelid heavy-chain antibodies (VHH) was isolated from a set of GFP-binding nanobodies and characterized as an enhancer of wild-type avGFP fluorescence [117]. This VHH domain was tested in combination with the tripartite complementation system. Co-localization experiments indicated that VHH does not bind GFP1–9 when expressed alone but do bind the reconstituted GFP while inducing a 5–8 fold increase in fluorescence [92]. The mechanism of action of this VHH-enhancing fluorescence nanobody remains poorly understood. One plausible explanation is that the antibody stabilizes interactions between residues required for chromophore formation and stabilization, thus improving the maturation rate upon binding. In-depth analysis of the crystal structure and spectral properties of the reconstituted tripartite split-GFP in complex with VHH may provide a better understanding of the mechanism underlying fluorescence modulation.

## Abbreviations

ASAP1	Accelerated Sensor of Action Potentials
AvGFP	*Aequorea victoria* Green Fluorescent Protein
BiFC	Bimolecular Fluorescence Complementation
CALM	Complementation-Activated Light Microscopy
CRISPR	Clustered Regularly Interspaced Short Palindromic Repeats
ER	Endoplasmic Reticulum
FKBP	FK506 Binding Protein
FP	Fluorescent Protein
FRAP	Fluorescent Recovery After Photobleaching
FRB	FKBP-Rapamycin Binding domain
FRET	Förster Resonance Energy Transfer
GBD	GTPase Binding Domain
GEF	Guanine nucleotide Exchange Factor
ORF	Open Reading Frame
PCA	Protein Complementation Assay
POI	Protein Of Interest
PPI	Protein-Protein Interaction
RAGE	Receptor for Advanced Glycation End product
RAP	Rapamycin
RBD	RHO Binding Domain
SPR	Surface Plasmon Resonance
SRTA	Sortase A
TetFC	Tetramolecular Fluorescence Complementation
TFIIH	Transcription Factor II H
TTDA	TrichoThioDystrophy-A
Tth	Thermus thermophiles
TriFC	Trimolecular Fluorescence Complementation
Y2H	Yeast-two-hybrid
